# Enhanced prognostic and immunomodulatory effects of novel cuproptosis-related long noncoding RNAs in wilms tumor

**DOI:** 10.3389/fmolb.2025.1566551

**Published:** 2025-05-27

**Authors:** Yadong Li, Mingjie Li

**Affiliations:** ^1^ Department of Clinical Laboratory, Fujian Maternity and Child Health Hospital College of Clinical Medicine for Obstetrics and Gynecology and Pediatrics, Fujian Medical University, Fuzhou, Fujian, China; ^2^ Department of Laboratory Medicine, Fujian Medical University Union Hospital, Fujian Medical University, Fuzhou, Fujian, China

**Keywords:** wilms tumor, cuproptosis, lncRNA, prognosis, immunology

## Abstract

**Objective:**

This study aims to develop a model for long non-coding RNA (lncRNA) associated with cuproptosis and assess the efficacy of immunotherapy and chemotherapy in children with Wilms tumor (WT) based on individualized risk scores.

**Methods:**

Data was obtained from the online database. Cox proportional hazards analysis and LASSO Cox regression were employed to generate cuproptosis-related lncRNA signatures. Patients were classified into high- and low-risk groups and clinical outcomes were further analysed. Tumor mutation burden and immunoinfiltration were calculated and potential immunotherapy response was evaluated. The sensitivity of immunotherapy and chemotherapy was ultimately analyzed based on individual risk scores associated with cuproptosis.

**Results:**

A eight cuproptosis-related lncRNAs signature was established and high-risk group showed a worse prognosis than the low-risk group. This model showed a good diagnostic performance. Low-risk group displayed an elevated tumor immune dysfunction and was more sensitive to 13 drugs.

**Conclusion:**

The current study introduces a novel approach for predicting clinical prognosis and determining the appropriate therapy for patients with WT.

## 1 Introduction

Wilms tumor (WT), the most common genitourinary tract cancer in the pediatric population, accounts for approximately 5% of all pediatric cancers (Tang et al., 2021). Over the past 50 years, the 5-year overall survival (OS) rate of WT in developed countries surpass 90% thanks to the concerted efforts of surgery, chemotherapy, and radiotherapy. However, event survival rates for children with certain disease types, such as undifferentiated pathological tumors, recurrent tumors, bilateral nephroblastomas, and unilateral high-risk nephroblastomas, remains low, with serious long-term complications (Gratias et al., 2016; Wong et al., 2016). All known mutations to date account for up to 50% of WT cases (Zhuo et al., 2021). Therefore, clarifying the molecular pathways of metastasis and progression while identifying novel treatment targets for WT survival and prognosis is significant.

The most recent studies on the mechanisms have demonstrated that cancer patients have significantly higher amounts of copper in tissues, serum, and cells (Blockhuys et al., 2017; Bian et al., 2022). Cuproptosis is a novel cell death pathway discovered by Tsvetkov et al. They found that lipoylated dihydrolipoamide S-acetyltransferase (DLAT) aggregation, which is related to mitochondrial tricarboxylic acids, may be attributed to an increase in intracellular copper. The tricarboxylic acid cycle promotes cell death and produces a proteotoxic stimulus (Tsvetkov et al., 2022). Moreover, recent findings demonstrated that cuproptosis was strongly correlated with various diseases, as well as cancer (Lv et al., 2022; Mangalmurti and Lukens, 2022; Moos et al., 2022). For this reason, identifying cancer-related cuproptosis biomarkers that can be used to facilitate improvement in patient progression and outcome prediction is of significance. Huang et al. successfully established a risk prediction model for WT through machine learning approaches and identified cuproptosis-related clusters encompassing five pivotal coding-genes. Notably, their investigation primarily centered on protein-coding genomic features (Huang et al., 2024).

Long noncoding RNAs (lncRNAs) represent a class of over 200 transcribed genomic elements that orchestrate gene regulation through transcriptional control, post-transcriptional modification, and epigenetic reprogramming (Jandura and Krause, 2017). The tumorigenic roles of lncRNAs have been extensively characterized across malignancies, with emerging evidence confirming their pathological significance in WT pathogenesis. Notably, Wang et al. (2024). Demonstrated that SNHG6 exhibits significant upregulation in both WT tissues and cellular models compared to normal controls. Mechanistically, this oncogenic lncRNA orchestrates multiple pro-tumorigenic processes by simultaneously promoting cellular proliferation, enhancing glycolytic metabolism, and suppressing apoptotic pathways. Complementing these findings, Zhang et al. (2019b). Identified SOX21-AS1 as another dysregulated lncRNA showing marked overexpression in WT specimens relative to adjacent non-tumor tissues and embryonic renal cells. Clinically, elevated SOX21-AS1 expression correlates with aggressive disease features, including larger tumor dimensions, advanced-stage presentation, and unfavorable histological subtypes. Functionally, experimental silencing of this lncRNA not only attenuated proliferative capacity and colony formation efficiency but also induced G1/S phase arrest through p57-mediated cell cycle regulation.

Although oncogenic roles of lncRNA are well-documented, the interplay between lncRNAs and cuproptosis remains characterized (Evans et al., 2016). Emerging studies have established that lncRNAs not only critically regulate tumor immune microenvironments through immune cell infiltration modulation but also orchestrate cytokine networks and checkpoint molecule expression. Furthermore, accumulating evidence demonstrates that specific lncRNAs create immunosuppressive niches via dual mechanisms: epigenetic remodeling of stromal components and direct lymphocyte interactions, thereby positioning them as promising immunotherapeutic targets. Building upon this mechanism, Li et al. (2024) demonstrated that cuproptosis-related lncRNAs may regulate tumor immune surveillance escape through immune infiltration-associated molecular pathways in lung cancer. Concurrently, independent investigations have revealed that elevated intratumoral copper ion concentrations promote PD-L1 upregulation through the HIF-1α/PD-L1 axis, whereas copper chelator administration effectively counteracts this immune evasion phenomenon in esophageal cancer (Sun et al., 2024).

Despite growing interest in cuproptosis regulation, the mechanistic roles of lncRNAs in WT pathogenesis remain underexplored. Key outstanding challenges in this area encompass: (1) Inadequate utilization of TARGET and TCGA databases for identifying WT-associated lncRNA signatures, (2) Unresolved clinical applicability of cuproptosis-related lncRNAs as diagnostic biomarkers, and (3) Uncharacterized interplay between lncRNA-modulated cuproptosis pathways and tumor microenvironment. To systematically bridge these knowledge gaps, we conducted a comprehensive multi-omics study integrating: (1) LASSO-based machine learning algorithms for prognostic signature development, (2) Multidimensional immune profiling combining ESTIMATE, CIBERSORT, MCP-counter and Xcell analytical frameworks, and (3) Predictive modeling of immunotherapy responsiveness through TIDE computational platform.

Our systematic investigation uncovered clinically relevant lncRNA signatures that modulate cuproptosis pathways, thereby shaping immuno-microenvironments in Wilms tumor. These findings elucidate the mechanistic underpinnings of copper metabolism dysregulation during tumor progression and highlight potential novel therapeutic targets for Wilms tumor.

## 2 Materials and methods

### 2.1 Data gathering and handling

The TARGET database was utilized to acquire additional human expressed and medical data for WT, which were subsequently made accessible on the TCGA data portal (https://portal.gdc.cancer.gov/projects/TARGET-WT, accessed 10 Feb 2025), encompassing a cohort of 125 WT patients. The procedure is succinctly delineated as follows. In this study, the TPM (Transcripts per million) expression profile data of TARGET-WT (Wilms Tumor) was downloaded from the TCGA official website. The probe IDs (ensemble ID) in the expression profile were converted into protein-coding mRNA and lncRNA using gencode v36 annotation file. After obtaining the expression spectrum, if an mRNA corresponds to multiple probes, the average value of these probes is considered as the expression level for this mRNA. During data partitioning, this study employed the createDataPartition function from R package caret to randomly allocate all data into training and validation sets at a 1:1 ratio. Table 1 provides a comprehensive summary of the participants’ clinical characteristics.

**TABLE 1:** Clinical features of 125 patients with Wilms tumor.

Clinical features	Patients (n = 125)	
	Number	Percentage (%)
Gender		
Female	71	56.8
Male	54	43.2
Race		
White	92	73.6
Non-White	33	26.4
Age (years)		
<5	78	62.4
≥5	47	37.6
Survival status		
Alive	75	60
Dead	50	40
Histologic classification		
FHWT	83	66.4
DAWT	42	33.6
Clinical stage		
Stage I	16	12.8
Stage Ⅱ	49	39.2
Stage Ⅲ	46	36.8
Stage Ⅳ	14	11.2

Notes: FHWT, Favorable Histology Wilms Tumor. DAWT, diffuse anaplastic wilms tumor.

### 2.2 Generation of cuproptosis-related lncRNAs

An extensive literature search was performed to identify protein-coding genes related to cuproptosis (Polishchuk et al., 2019; Aubert et al., 2020; Hong et al., 2020; Yi et al., 2020; Dong et al., 2021; Ren et al., 2021; Xu F. et al., 2021; Kahlson and Dixon, 2022; Tsvetkov et al., 2022). The fundamental functionalities and literature references of the 19 cuproptosis-associated genes can be found in Supplementary Table S1. The relationship between genes and lncRNAs associated with cuproptosis was then evaluated using the Spearman correlation analysis. Only coefficients above 0.4 with p-values less than 0.01 were considered significant.

### 2.3 Cuproptosis-related lncRNA signature characterization and verification

The study enrolled 125 patients and assigned them randomly to the testing and training groups. The LASSO Cox regression method was utilized to establish the lncRNA signature linked with cuproptosis. The following formula was used to generate the risk score: Risk score = (expression_lncRNA1_ × β_lncRNA1_) + (expression_lncRNA2_ × β_lncRNA2_) + . + (expression _lncRNA n_ × β _lncRNA n_). The testing group, training group, and entire cohort were then categorized into a high- and low-risk group based on their median risk scores. Variations in OS between the low- and high-risk groups were verified using the Kaplan-Meier analysis.

Furthermore, we conducted an in-depth analysis of the clinical characteristics and identified significant independent predictors using Cox regression analysis. Additionally, we developed a personalized clinical signature and nomogram based on lncRNAs associated with cuproptosis to accurately estimate the 1-, 3-, and 5-year survival rates in individuals with WT. Finally, we validated the predictive efficacy of the nomogram through calibration curve analysis and assessment of consistency index (C-index).

### 2.4 Construction and functional enrichment analysis of the lncRNA–mRNA co-expression network

The correlation between cuproptosis-related lncRNAs and mRNA expression was assessed using the Pearson correlation coefficient (|r| > 0.3, p < 0.001). Additionally, R software with clusterProfiler packages (Yu et al., 2012) was employed to visualize the lncRNA-mRNA co-expression network. Furthermore, Gene Ontology (GO) functional enrichment analysis and Kyoto Encyclopedia of Genes and Genomes (KEGG) pathway analysis were performed, with visualization achieved through R software utilizing the limma package.

### 2.5 Tumor mutation burden (TMB) analyses

The quantity of nonsynonymous point mutations in somatic cells was also calculated and shown for each sample using the R package maftools (Xu Q. et al., 2021) and TMB used nonsynonymous points and code-switching inconsistencies with a detection threshold of 5%. Next, survival curves and risk scores for TMB were derived by comparing the low- and high-risk groups.

### 2.6 Profiling of immunology characteristics

The “readxl” package (https://cran.r-project.org/web/packages/readxl/index.html) in R was utilized to evaluate immune cells and responses in the two groups. The ESTIMATE, CIBERSORT, MCPcounter (Lin et al., 2024), and xcell procedures were employed to determine the percentage of each immune cell type in all individuals belonging to the low and high-risk groups. Activity level for the 13 immunity-related functions was determined, and differences between both groups were compared using the “GSVA” package in R. Similarly, Pearson correlation analysis was used to characterize the connection between cuproptosis-linked lncRNAs and immune checkpoints.

### 2.7 Drug sensitivity analysis

Evaluating the response to immunotherapy in WT patients using the Tumor Immune Dysfunction and Exclusion (TIDE) framework, available at http://tide.dfci.harvard.edu, can assist physicians in identifying patients who are more likely to derive benefit from immunotherapy. The ‘vroom, rio’ package was utilized for visualizing the TIDE scores of both low- and high-risk groups. Additionally, leveraging the Genomics of Cancer Drug Sensitivity database (GDSC), accessible at http://www.cancerrxgene.org/, we employed the pRRophetic software package to evaluate drug IC50 values and predict sensitivity for chemotherapy with statistical significance determined by P values <0.05. This analysis accounted for variations in drug sensitivity between low- and high-risk groups.

### 2.8 Statistical analysis

The Wilcoxon rank-sum test was employed to determine differences between the low- and high-risk groups. All statistical analyses were conducted using R (version 4.1.2), with a significance level of P < 0.05, unless otherwise specified. The survminer package in R was utilized to estimate overall survival for the two groups, while Cox regression analysis was performed to assess survival rates. Time-dependent receiver operating characteristic (ROC) curves were generated using the time ROC package in R. Heatmap was constructed using the heatmap package, and data visualization was accomplished using ggplot2 (V4.1.2).

## 3 Results

### 3.1 Cuproptosis-related lncRNA recognition with predictive significance

We identified a total of 125 wild-type samples, from which we obtained 19,938 mRNAs and 16,877 lncRNAs. Furthermore, we compiled a curated list of 19 protein-coding genes (NLRP3, DLAT, NFE2L2, ATP7B, FDX1, ATP7A, SLC31A1, LIAS, GCSH, LIPT1, PDHA1, GLS, LIPT2, CDKN2A,DLD,MTF1, PDHB, DBT and DLST) that are closely associated with cuproptosis.

### 3.2 Production of the cuproptosis-related lncRNA landscape

**FIGURE 1:**
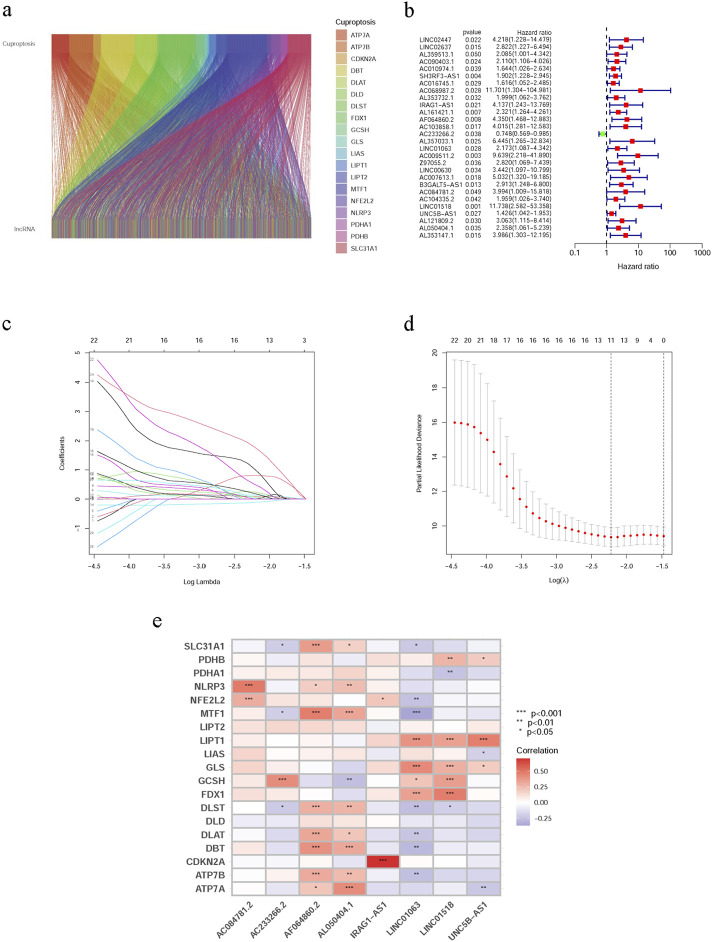
Construction of the cuproptosis-associated lncRNA model. **(a)** The relationship between mRNAs and cuproptosis-related lncRNAs was demonstrated using the Sankey Diagram. **(b)** The Forest plot displays the 28 lncRNAs with hazards ratios (95% confidence intervals) and p values for their correlation with Wilms tumor prognosis based on univariate Cox proportional hazards analysis. **(c)** Coefficients for cuproptosis-related lncRNAs were calculated using LASSO regression. The corresponding risk coefficients (β) are presented below. IRAG1-AS1 (3.30), AF064860.2 (1.02), AC233266.2 (0.42), LINC01063 (0.90), AC084781.2 (1.74), LINC01518 (3.93), UNC5BAS1 (0.40), and AL050404.1 (0.83). **(d)** Confidence intervals at each lambda. **(e)** The association between the eight predictive cuproptosis-linked lncRNAs and the 19 cuproptosis-associated genes was presented using a heat plot.

This study included a total of 125 patients with WT, out of which 63 were randomly assigned to the training group, while the remaining patients were allocated to the testing group through randomization. Table 1 presents an overview of the clinical characteristics of the included individuals with WT. Importantly, no significant differences in clinical characteristics were observed between the testing and training groups (Supplementary Table S2). We compiled a list of 19 genes associated with cuproptosis based on literature review and GO annotations. Spearman correlation analysis identified 1,277 lncRNAs related to cuproptosis that showed correlation with cuproptosis-related genes (Figure 1A).

The identification of cuproptosis-related lncRNAs was accomplished through a two-step analysis. Firstly, univariate Cox regression analysis was conducted in the training group, resulting in the identification of 28 lncRNAs significantly associated with prognosis (Figure 1B). Secondly, to address multicollinearity, LASSO Cox regression analysis was performed to select predictive features, leading to the extraction of eight lncRNAs from the previously identified set of 28 cuproptosis-related lncRNAs (Figures 1C, D), namely, IRAG1-AS1, AF064860.2, AC233266.2, LINC01063, AC084781.2, LINC01518, UNC5B-AS1 and AL050404.1. The association between these eight lncRNAs and the set of 19 cuproptosis-related genes is depicted in Figure 1e.

According to their respective gene risk coefficients (β) and expression levels, the risk scores for each lncRNA were calculated as follows: IRAG1-AS1*3.30054925,544,454, AF064860.2*1.01850795,166,343, AC233266.2*−0.415315455,163,119, LINC01063*0.901425676,879,008, AC084781.2*1.74313414,998,208, LINC01518*3.93205970,154,075, UNC5BAS1*0.404293992,468,113, and AL050404.1*0.82931678,500,327. These eight cuproptosis-related lncRNAs exhibited strong correlations with other genes related to cuproptosis within our study cohort. For instance, AL050404.1 was positively correlated with ATP7B, ATP7A, etc., but negatively correlated with GCSH. Whereas, LINC01063 was positively correlated with GLS,LIPT1, etc., but negatively correlated with DLAT, DBT, etc.

### 3.3 Authenticating the lncRNA signature associated with cuproptosis

**FIGURE 2:**
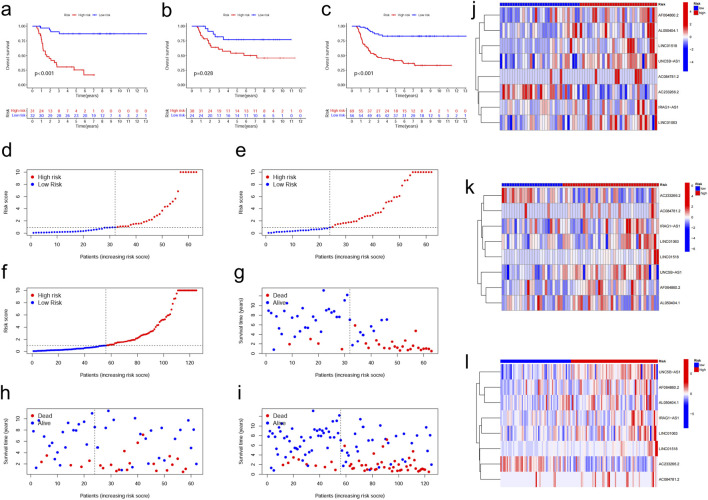
A risk prediction model for subjects with Wilms tumor. **(a–c)** Overall survival was compared between the high- and low-risk groups in the training group, testing group, and entire cohort. **(d–f)** Risk curves were created based on the risk scores of each sample from the training group, testing group, and entire cohort. **(g–i)** Scatter plots were created according to each sample survival status in the training group, testing group, and entire cohort. **(j–l)** Expression heat maps for the eight cuproptosis-related lncRNAs were shown for the testing group, training group, and entire cohort.

To validate the accuracy and prognostic value of the cuproptosis-related lncRNA signature, the training group was stratified into high-risk (n = 31) and low-risk (n = 32) groups based on the median risk score. To evaluate the predictive accuracy of the model, Kaplan-Meier (K-M) survival curves were constructed to compare overall survival (OS) between different risk groups. The results revealed that the model exhibited high predictive accuracy, with statistically significant differences in survival outcomes observed between the groups (training cohort: p = 0.028; test cohort and entire cohort: p < 0.001; Figures 2A–C). As depicted in Figures 2D–F, risk curves presenting the distribution of risk scores across the training, testing, and entire cohorts indicated a significantly higher proportion of deceased patients in the high-risk group compared to the low-risk group. Scatter plots (Figures 2G–I) were generated based on the survival status of each sample within the training, testing, and overall cohorts. Furthermore, heatmaps illustrated the expression patterns of the eight cuproptosis-related lncRNAs associated with the risk model in both high-risk and low-risk cohorts (Figures 2J–L). To identify differences between high- and low-risk individuals, principal component analysis was conducted using the “scatterplot3d” and “ggplot2” packages for R. The results of our study revealed a distinct disparity in the gene expression profile between the high- and low-risk cohorts (Supplementary Figure S1).

### 3.4 Development of nomograms for individualized prognosis prediction

**FIGURE 3:**
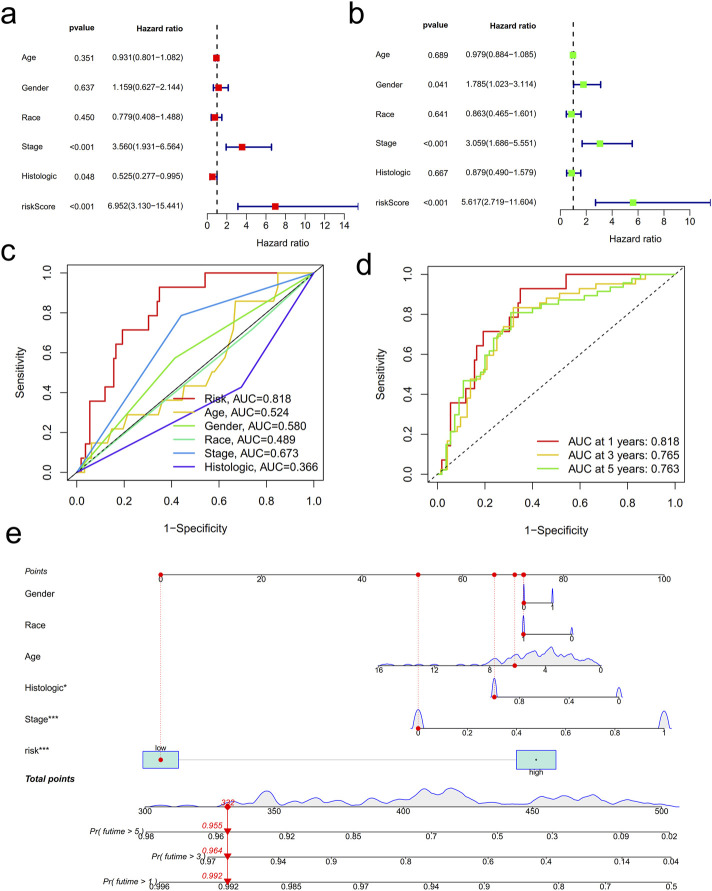
Clinical implications of cuproptosis-related lncRNAs in Wilms tumor. **(a)** Multivariate and **(b)** univariate Cox proportional hazards analysis to determine whether cuproptosis-related lncRNA risk scores were independent predictors of prognosis. **(c)** Receiver operating characteristic (ROC) curves for clinical characteristics and signature-based risk scores. **(d)** ROC curves were used to verify the prognostic value of the risk scores for all cases. **(e)** A nomogram combining both clinicopathological parameters and risk scores to predict the 1-, 3-, and 5-year survival of individuals with WT.

To determine whether the lncRNA signature associated with cuproptosis could function as an independent prognostic indicator in patients with Wilms tumor, we conducted multivariate and univariate Cox regression analyses. The results of the multivariate Cox regression analysis revealed that cancer stage, histologic score, and risk score were robust predictors of prognosis in individuals with WT (Figure 3A). Furthermore, the univariate Cox regression analysis demonstrated that sex, risk score, and cancer stage independently predicted prognosis among subjects with WT (Figure 3B). Moreover, the ROC curves illustrated that the risk score exhibited a significantly higher area under the curve value (0.818) compared to other clinical characteristics (Figure 3C). Notably, the lncRNA signature associated with cuproptosis displayed substantial predictive value at 1-, 3-, and 5-year time points, as evidenced by ROC values of 0.818, 0.765, and 0.713 respectively (Figure 3D). In order to enhance the accuracy of current prognostic models, we developed a nomogram tool based on the relationship between clinical characteristics and the cuproptosis-associated lncRNA signature. As depicted in Figure 3E and Supplementary Figure S2, this model generated a nomogram with a c-index of 0.727 which facilitated predictions regarding survival rates at 1-, 3-, and 5-year time points for individuals diagnosed with WT.

### 3.5 LncRNA–mRNA co-expression network construction

The lncRNA-mRNA network was constructed to investigate the potential involvement of lncRNAs associated with curoptosis in WT tumors. Notably, this network comprised 404 lncRNA-mRNA pairings involving eight distinct lncRNAs and 400 unique mRNAs (Supplementary Figure S3A). Subsequently, the GO analysis revealed significant enrichment of cellular components in the ‘DNA packaging complex’, biological processes in the ‘cytokine-mediated signaling pathway’, and molecular function primarily in ‘protein heterodimerization activity’ (Supplementary Figure S3B). Moreover, KEGG pathway analysis demonstrated that the top five enriched pathways included cytosolic DNA-sensing, lysine degradation, RIG-I-like receptor, NF-kappa B, and RIG-I-like receptor signaling pathways (Supplementary Figure S3C).

### 3.6 Relationship between the risk score of cuproptosis-related lncRNA signature and TMB

**FIGURE 4:**
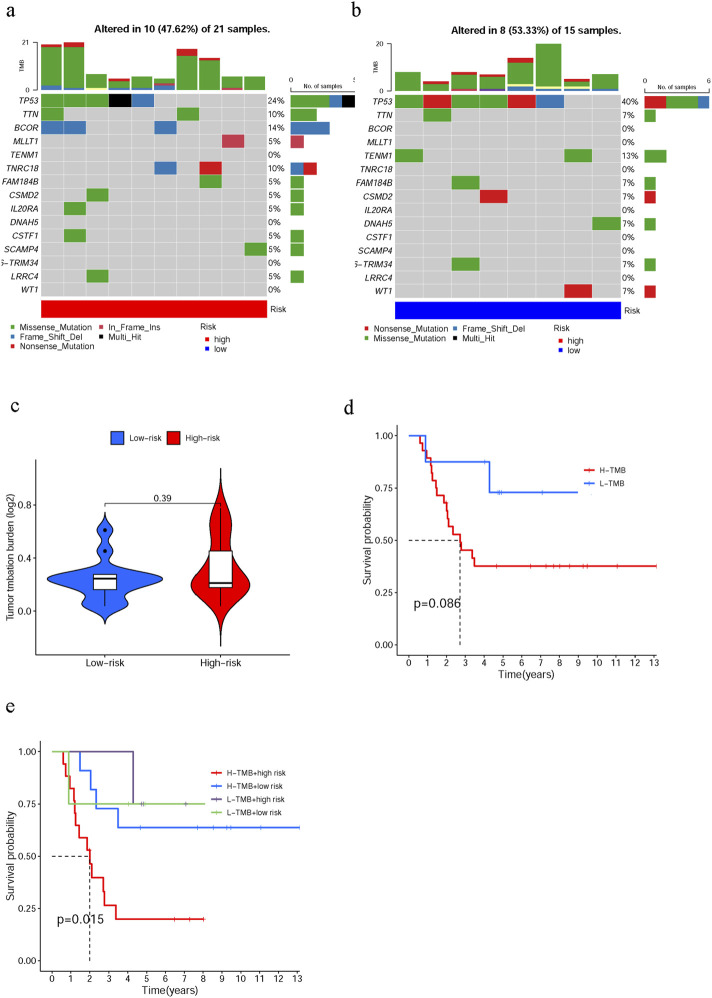
Link between the cuproptosis-related lncRNA risk score and tumor mutation burden (TMB). The waterfall plot revealed the somatic mutations in the 15 most important genes in the high- **(a)** and low-risk **(b)** groups. **(c)** The TMB was compared between the high- and low-risk groups. **(d)** Kaplan–Meier curves for both elevated and reduced TMB groups. **(e)** Kaplan–Meier curves stratified according to TMB and risk score across subgroups.

The somatic mutation profiles were analyzed in both the low- and high-risk groups. Notably, a significant increase in the frequency of somatic mutations was observed for BCOR (14% vs 0%) and TTN (10% vs 7%) in the high-risk group (Figure 4A), whereas an elevated occurrence of somatic mutations was found for TP53 (24% vs 40%) and WT1 (0% vs 7%) in the low-risk group (Figure 4B). Furthermore, there was no discernible difference in tumor mutation burden (TMB) between the high- and low-risk groups (Figure 4C), while survival time did not significantly differ between individuals with higher or lower TMB levels (p = 0.086, Figure 4D). However, the prognosis deteriorated in the high-risk group as TMB increased, underscoring a significant synergy between these two measures (p = 0.015, Figure 4E).

### 3.7 Correlation analysis of different groups and immunologic characteristics

**FIGURE 5:**
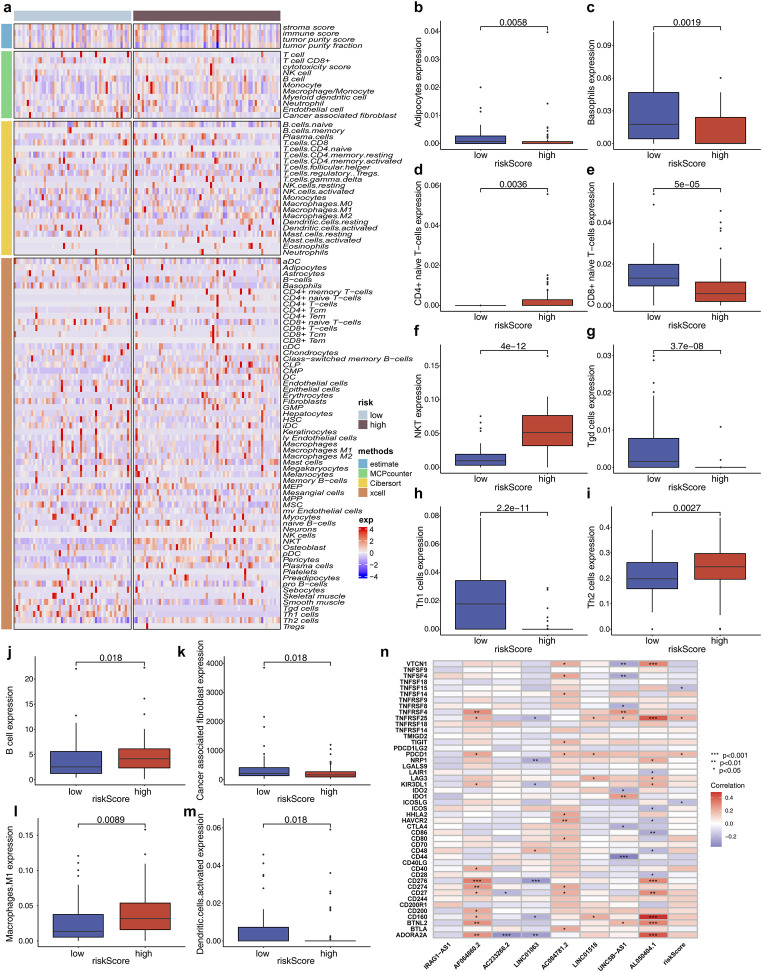
Immune-related characteristics of the high- and low-risk groups. **(a)** Heat map showing the immune status of the low- and high-risk groups according to ESTIMATE, MCPcounter, CIBERSORT, and xcell algorithms. **(b–m)** Comparison of immune cell infiltration between the low- and high-risk groups. **(n)** Relationship between cuproptosis-associated lncRNAs and risk scores and immune checkpoint.

The immune infiltration level in the low- and high-risk groups was evaluated using ESTIMATE, MCPcounter, CIBERSORT, and xcell algorithms (Figure 5A). The results demonstrated an enrichment of adipocytes, CD8+-naïve T cells, basophils, Tgd cells, Th1 cells, dendritic cells, and cancer-related fibroblasts in the low-risk group (all p < 0.05, Figures 5B–H). NKT cells, CD4+-naïve T cells, Th2 cells, B cells, and M1 macrophages were found to be concentrated in the high-risk group (all p < 0.05, Figures 5I–M). Moreover, the expression levels of TNFSF15, TNFRSF25, PDCD1, and ICOSLG immune checkpoint molecules were relatively higher in the high-risk group (Figure 5N).

### 3.8 Drug sensitivity analysis

**FIGURE 6:**
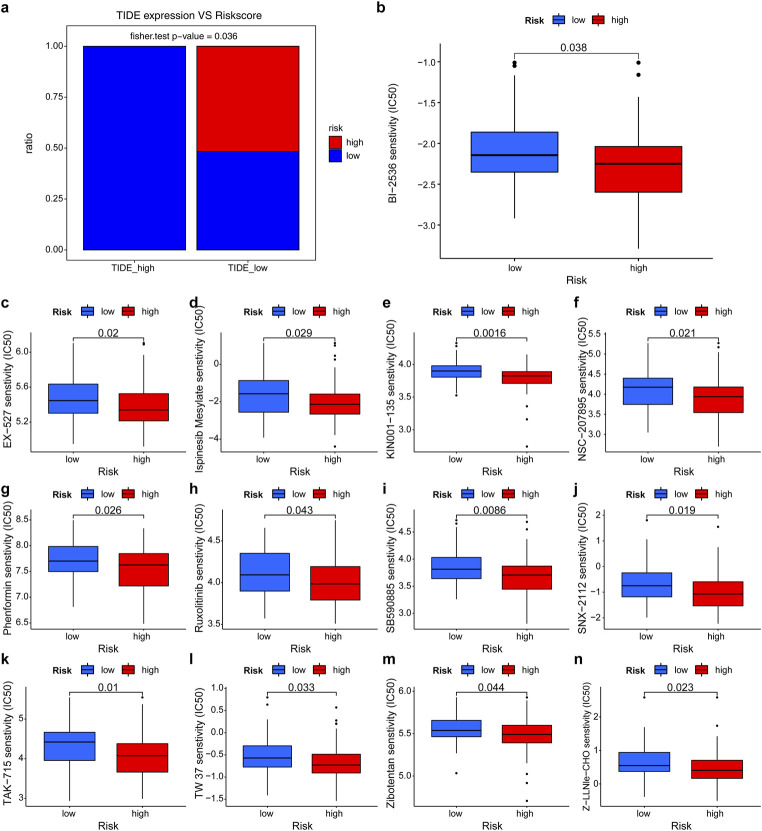
The role of the prognostic risk scoring model in chemotherapy. **(a)** Comparison of TIDE scores between the high - and low-risk groups. **(b–n)** Comparison of sensitivity to chemotherapeutic agent (IC50) between the high- and low-risk groups. The lower the IC50 value, the greater the efficacy in inducing tumor cell death. Notes: BI-2536 (Polo-like kinase I inhibitor), EX-527(Sirtuin one inhibitor), KIN001-135 (PI3K inhibitor), Ispinesib Mesylate (KIF11 inhibitor), NSC-207895 (XIAP inhibitor), Phenformin (Mitochondrial Complex I inhibitor), Ruxolitinib (Janus kinase one and two inhibitor), SB590885 (BRAF inhibitor), SNX-2112 (HSP90 inhibitor), TAK-715 (p38 MAPK inhibitor), TW37 (Bcl-2 inhibitor), Zibotentan (Endothelin Receptor A inhibitor), Z-LLNle-CHO (Proteasome inhibitor).

A comparison of the TIDE scores between both groups found that the low-risk group had significantly higher scores than the high-risk group (p = 0.036, Figure 6A). More importantly, this indicated that the low-risk group had a weaker immune response to treatment, as well as a large possibility for cancer immune escape. To further investigate the impact of risk scores on treatment outcomes in patients with WT, we conducted a study evaluating treatment response in high-risk and low-risk WT patients. Accordingly, we discovered that individuals with high- and low-risk WT exhibited significant variations in the estimated IC50 values of the 13 therapeutic agents and that subjects with high-risk exhibited increased sensitivity to all 13 therapeutic agents (The lower the IC50 value, the greater the efficacy in inducing tumor cell death, all p < 0.05, Figures 6B–N).

## 4 Discussions

Despite being the most prevalent pediatric renal cancer, Wilms tumor (WT) or nephroblastoma can be effectively managed following the standard treatment protocol outlined in the International Society of Pediatric Nephrology guidelines. This comprehensive approach includes preoperative chemotherapy, surgical intervention, and postoperative care. However, it is concerning that 15% of children still experience relapses, with a majority occurring within 2 years of initial diagnosis. Furthermore, the survival rate for recurrent cases stands at 50%, primarily influenced by specific high-risk factors such as bilateral WT, disease types associated with unfavorable prognosis, advanced tumor stage, and prior treatments (Spreafico et al., 2009; Pritchard-Jones et al., 2012; Malogolowkin et al., 2013; Brok et al., 2016; Brok et al., 2018). Therefore, it is imperative to identify prospective prognostic markers and molecular characteristics unique to WT patients in order to optimize patient outcomes.

As a trace element in the human body, copper facilitates the function of various copper-dependent enzymes, such as superoxide dismutase 1 (Cu/Zn-SOD), ceruloplasmin (CP), cytochrome oxidase (Cox), and angiopoietin (Tapiero et al., 2003). The active homeostasis mechanism maintains cellular copper ion levels at extremely low concentrations. When accumulated intracellularly, copper plays a vital role in mammalian physiological processes including iron absorption, redox chemistry, free radical scavenging, mitochondrial respiration, and elastin cross-linking. However, exceeding a certain threshold renders copper toxic and induces cell death (Liu S. J. et al., 2021). Cuproptosis was initially identified by Todd R. Golub (Tsvetkov et al., 2022), who elucidated the involvement of disrupted copper metabolism and mitochondrial imbalances in cell demise. Nevertheless, the precise contribution of cuproptosis to WT remains unclear. Therefore, screening for genes associated with cuproptosis and identifying biomarkers capable of predicting responses to immunotherapy and chemotherapy represent promising strategies for enhancing therapeutic efficacy and reducing mortality rates in WT.

According to recent studies, lncRNAs are likely to play a pivotal role in regulating key components of the cuproptosis pathway. Specifically, these lncRNAs may modulate intracellular copper homeostasis by regulating the expression of copper transporters such as CTR1 and ATP7A/B, which are crucial for maintaining appropriate cellular copper levels. Dysregulation of these transporters can lead to copper ion accumulation, thereby causing mitochondrial dysfunction and ultimately inducing cell death. Moreover, lncRNAs may also regulate genes involved in mitochondrial respiration and reactive oxygen species (ROS) production. The excessive ROS generated by mitochondria can further exacerbate cellular damage and trigger cuproptosis, consequently affecting tumor cell growth (Bhat et al., 2024).

The TCGA database serves as a robust and comprehensive resource for tumor research. Luo Y et al. utilized this database to investigate RNA modification-related genes in breast cancer, identifying WDR4 as a critical factor contributing to the RMscore. The inhibition of WDR4 was shown to significantly impair breast cancer progression both *in vitro* and *in vivo* (Luo et al., 2024). Additionally, Tian W leveraged this database to uncover a novel gene signature in breast cancer that not only accurately predicts clinical prognosis and metastasis but also offers strong support for personalized clinical decision-making. Our findings not only provide a valuable reference for personalized medicine but also underscore the reliability of the TCGA database in facilitating biomarker discovery and mechanistic exploration (Tian et al., 2023).

Previous studies have suggested that aberrant lncRNA expression may facilitate the development and progression of cancer (Da et al., 2021; Liu S. J. et al., 2021; Meng et al., 2022). However, this study is the first comprehensive investigation into the involvement of lncRNAs associated with cuproptosis in WT. In this study, we identified eight cuproptosis-related lncRNAs (IRAG1-AS1, AF064860.2, AC233266.2, LINC01063, AC084781.2, LINC01518, UNC5B-AS1, and AL050404.1) through LASSO regression and multivariate Cox regression analysis (Figure 1). Among these, UNC5B-AS1 was found to be a risk factor for poor osteosarcoma prognosis (Yang et al., 2022), promoting liver cancer cell migration, growth, and epithelial-mesenchymal transition (Huang et al., 2021), as well as exhibiting high expression levels in papillary thyroid carcinoma (Kim et al., 2022). Furthermore, LINC01063 has been identified as an oncogene in melanoma (Xu et al., 2022) and an autophagy-associated lncRNA capable of predicting colorectal tumor prognosis (Duan et al., 2022), while LINC01518 has been shown to function as an endogenous competing RNA in esophageal squamous cell carcinoma (Zhang D. et al., 2019). To date, no detailed investigations have been conducted on the remaining five cuproptosis-related lncRNAs. In order to categorize individuals into low-risk or high-risk groups, a risk score was calculated based on their genetic traits. Our findings suggest that the established risk score serves as an independent prognostic factor for patients with WT. Survival analysis revealed significantly poorer outcomes in the high-risk group compared to the low-risk group (Figure 2).

After establishing the initial prospective model for cuproptosis-related analysis, we determined that the risk score serves as an accurate indicator of patient prognosis and overall survival (OS). Our findings from the ROC curve demonstrate that cuproptosis-related long non-coding RNAs possess sufficient predictive utility in evaluating OS among WT patients, surpassing ferroptosis-related lncRNAs (Liu H. et al., 2021). Furthermore, our nomogram risk assessment of this prognostic risk score validates our model. The implementation of our nomogram may enable physicians to tailor monitoring strategies specific to each patient with WT, thereby advancing medical interventions to improve clinical outcomes.

The tumor mutation burden (TMB) is a known factor that has a significant correlation with immunotherapy. A study demonstrated that high TMB scores were indicative of favorable immunotherapy outcomes in multiple tumor cases (Liu et al., 2022). However, our findings did not show any notable variation in TMB between the high- and low-risk groups. This could potentially be attributed to the limited sample size of the aforementioned studies. Interestingly, when integrating TMB with risk scores, we discovered that individuals in the elevated TMB and high-risk group exhibited the worst survival rates (Figure 4E), suggesting that risk factors have a crucial impact on patient survival. Another study revealed that Tumor Immune Dysfunction and Exclusion (TIDE) was a superior method for modeling tumor immune escape and effectively predicting the immunotherapeutic impact of malignancies (Sha et al., 2022). The present study represented the first attempt to compare TIDE scores between low- and high-risk cohorts. The unexpected inverse correlation observed between risk scores and TIDE scores further suggests that individuals at higher risk may derive greater benefits from immunotherapy.

Evidence indicates that copper plays a pivotal role in immune regulation (St Paul and Ohashi, 2020; Culbertson and Culotta, 2021; Raskov et al., 2021). The infiltration of cells may enhance our empirical understanding of the outcomes of antitumor treatment in WT, thereby facilitating more effective immunotherapy techniques to elucidate the function of cuproptosis in the tumor microenvironment (TME). In this study, the low-risk group demonstrated increased infiltration of immune cells, such as CD8+T cells, adipocytes, basophils, Tgd cells, Th1 cells, cancer-associated fibroblasts, and activated dendritic cells, compared to the high-risk group, indicating a stronger anti-cancer immune response. The low-risk group exhibited enhanced infiltration of immune cells, including CD8^+^ T cells, adipocytes, basophils, Tgd cells, Th1 cells, cancer-associated fibroblasts, and activated dendritic cells when compared to the high-risk group (Figure 5). This suggests a more robust anti-cancer immune response.

The presence of high concentrations of Th1 cells in the tumor microenvironment also contributes to a favorable prognosis (Fridman et al., 2012). Our findings indicate that the high-risk group exhibited elevated levels of NKT cells, CD4^+^ T cells, Th2 cells, B cells, and M1 macrophages. It is worth noting that B cells can exhibit both anti-cancer and cancer-promoting properties simultaneously, highlighting their heterogeneity (Sharonov et al., 2020). Several types of tumors with enriched NK cell populations such as gastric, colorectal, pulmonary, hepatic, and urinary system cancers often demonstrate improved prognoses. However, the effectiveness of NK cell activity within the tumor microenvironment is frequently limited (Terrén et al., 2019). The results from our study further support this conclusion. The findings suggest that the assessment of TMB, TIDE, and TME may serve as a more robust approach in predicting the response to immunotherapy.

In this study, we determined the IC50 curves of 13 anticancer drugs (BI-2536, EX-527, Ispinesib mesylate, KIN001-135, NSC-207895, Phenformin, Ruxolitinib, SB590885, SNX-2112, TAK-715) to predict their efficacy in pharmacotherapy (Figure 6). The IC50 values of all 13 drugs were found to be higher in the low-risk group compared to the high-risk group. Significant differences in drug sensitivity were observed between these risk groups. These findings provide valuable guidance for physicians when selecting drug agents based on risk scores.

In conclusion, we observed a significant association between cuproptosis-related lncRNA signatures and the prognosis of patients with WT. Furthermore, we assessed the relationship between establised risk scores and efficacy of chemotherapy and immunotherapy. The findings of this study may provide a novel exploratory approach to unraveling the cuproptosis pathway and expanding current insights into the treatment of WT patients.

## Data Availability

The datasets presented in this study can be found in online repositories. The names of the repository/repositories and accession number(s) can be found below: https://portal.gdc.cancer.gov/projects/TARGET-WT.

## Abbreviations

LncRNA, Long non-coding RNA; WT, Wilms tumor; OS, Overall survival; GO, Gene Ontology; KEGG, Kyoto Encyclopedia of Genes and Genomes; TMB, Tumor mutation burden; TIDE, Tumor Immune Dysfunction and Exclusion; GDSC, Genomics of Cancer Drug Sensitivity database; TME, Tumor microenvironment; ROC, Receiver operator characteristic curve; FHWT, Favorable Histology Wilms Tumor; DAWT, Diffuse Anaplastic Wilms Tumor.
