# Supplementary material for: Enhanced prognostic and immunomodulatory effects of novel cuproptosis-related long noncoding RNAs in wilms tumor
Source: Front Mol Biosci. 2025 May 27;12:1566551. doi: 10.3389/fmolb.2025.1566551 (PMC12148909; doi:10.3389/fmolb.2025.1566551)
Supplement: Supplementary file 1 [file Supplementaryfile1.docx]

**Supplementary Table 1.** The fundamental functionalities and literature references of the 19 cuproptosis-associated genes.

| Gene name | Gene function（from NCBI） | Literary sources |
| --- | --- | --- |
| ATP7B | This gene belongs to the P-type cation transport ATPase family and encodes a copper-transporting ATPase. It functions as a monomer to export copper from cells, such as hepatic copper into bile. Mutations in this gene are associated with Wilson disease, characterized by copper accumulation. | Polishchuk EV, et al. Gastroenterology. 2019;156: 1173-1189.e5. |
| ATP7A | This gene encodes a transmembrane protein responsible for copper transport across membranes. The protein is located in the trans Golgi network, supplying copper to copper-dependent enzymes in the secretory pathway | Holloway ZG, et al. Mol Biol Cell. 2013;24(11):1735-S8. |
| PDHA1 | The pyruvate dehydrogenase (PDH) complex, a nuclear-encoded mitochondrial multienzyme, converts pyruvate to acetyl-CoA and CO2, linking glycolysis to the tricarboxylic acid (TCA) cycle. | Tsvetkov P, et al. Science. 2022;375(6586):1254-1261. |
| PDHB | The function of PDHB is analogous to that of PDHA1, yet the protein subunit encoded by each gene differs. | Tsvetkov P, et al. Science. 2022;375(6586):1254-1261. |
| FDX1 | This gene encodes a small iron-sulfur protein that transfers electrons from NADPH through ferredoxin reductase to mitochondrial cytochrome P450, involved in steroid, vitamin D, and bile acid metabolism. | Tsvetkov P, et al. Science. 2022;375(6586):1254-1261. |
| LIAS | The protein encoded by this gene belongs to the biotin and lipoic acid synthetases family. Localized in the mitochondrion, this iron-sulfur enzyme catalyzes the final step in the de novo pathway for the biosynthesis of lipoic acid, a potent antioxidant. | Tsvetkov P, et al. Science. 2022;375(6586):1254-1261. |
| LIPT1 | The process of transferring lipoic acid to proteins involves two steps. First, lipoate-activating enzyme activates lipoic acid to form lipoyl-AMP. Second, the protein encoded by this gene transfers the lipoyl group to apoproteins. | Tsvetkov P, et al. Science. 2022;375(6586):1254-1261. |
| DLD | This gene encodes a class-I pyridine nucleotide-disulfide oxidoreductase family member. The protein functions as a dehydrogenase in homodimeric form, participating in energy metabolism complexes, and as a protease in monomeric form. Mutations in this gene cause E3-deficient maple syrup urine disease and lipoamide dehydrogenase deficiency. | Tsvetkov P, et al. Science. 2022;375(6586):1254-1261. |
| GCSH | Glycine degradation is performed by the glycine cleavage system, comprising four mitochondrial proteins: P protein (glycine decarboxylase), H protein (lipoic acid-containing), T protein (tetrahydrofolate-requiring), and L protein (lipoamide dehydrogenase). The gene encodes the H protein, which transfers glycine's methylamine group from P protein to T protein. Defects in this gene cause nonketotic hyperglycinemia (NKH). | Tsvetkov P, et al. Science. 2022;375(6586):1254-1261. |
| DLST | This gene encodes a mitochondrial protein from the 2-oxoacid dehydrogenase family. It is the E2 component of the 2-oxoglutarate dehydrogenase complex, which catalyzes the conversion of 2-oxoglutarate to succinyl-CoA and CO2. | Tsvetkov P, et al. Science. 2022;375(6586):1254-1261. |
| DLAT | This gene encodes the E2 component of the pyruvate dehydrogenase complex (PDC), which resides in the inner mitochondrial membrane and catalyzes the conversion of pyruvate to acetyl coenzyme A. | Tsvetkov P, et al. Science. 2022;375(6586):1254-1261. |
| SLC31A1 | The protein encoded by this gene is a high-affinity copper transporter found in the cell membrane. The encoded protein functions as a homotrimer to effect the uptake of dietary copper. | Tsvetkov P, et al. Science. 2022;375(6586):1254-1261. |
| LIPT2 | This gene encodes a mitochondrial protein that catalyzes the transfer of octanoic acid to lipoate-dependent enzymes such as octanoyl-ACP. | Tsvetkov P, et al. Science. 2022;375(6586):1254-1261. |
| GLS | This gene encodes the K-type mitochondrial glutaminase. The encoded protein is an phosphate-activated amidohydrolase that catalyzes the hydrolysis of glutamine to glutamate and ammonia. | Tsvetkov P, et al. Science. 2022;375(6586):1254-1261. |
| CDKN2A | This gene encode structurally related isoforms known to function as inhibitors of CDK4 kinase. Through the regulatory roles of CDK4 and p53 in cell cycle G1 progression, share a common functionality in cell cycle G1 control. This gene is frequently mutated or deleted in a wide variety of tumors, and is known to be an important tumor suppressor gene. | Tsvetkov P, et al. Science. 2022;375(6586):1254-1261. |
| MTF1 | This gene encodes a transcription factor that induces expression of metallothioneins and other genes involved in metal homeostasis in response to heavy metals such as cadmium, zinc, copper, and silver. | Tsvetkov P, et al. Science. 2022;375(6586):1254-1261. |
| DBT | The branched-chain alpha-keto acid dehydrogenase complex (BCKD) is an inner-mitochondrial enzyme complex involved in the breakdown of the branched-chain amino acids isoleucine, leucine, and valine. The BCKD complex is thought to be composed of a core of 24 transacylase (E2) subunits, and associated decarboxylase (E1), dehydrogenase (E3), and regulatory subunits. This gene encodes the transacylase (E2) subunit. | Tsvetkov P, et al. Science. 2022;375(6586):1254-1261. |
| NLRP 3 | This gene encodes a pyrin-like protein containing a pyrin domain, a nucleotide-binding site (NBS) domain, and a leucine-rich repeat (LRR) motif. This protein interacts with the apoptosis-associated speck-like protein PYCARD/ASC, which contains a caspase recruitment domain, and is a member of the NLRP3 inflammasome complex. This complex functions as an upstream activator of NF-kappaB signaling, and it plays a role in the regulation of inflammation, the immune response, and apoptosis. | DongJJ, et al. Cell Death Dis. 2021;12(1):87. |
| NRF2 | This gene encodes a transcription factor which is a member of a small family of basic leucine zipper (bZIP) proteins. The encoded transcription factor regulates genes which contain antioxidant response elements (ARE) in their promoters. | Ren XY, et al. Redox Biol. 2021;46:102122. |

**Supplementary Table 2.** Clinical characteristics between the test set and the training set.

| Clinical features | | Total | Test-group | Train- group | *p*-value |
| --- | --- | --- | --- | --- | --- |
| Gender | Female | 71 (56.8%) | 34 (54.8%) | 37 (58.7%) | 0.796 |
|  | Male | 54 (43.2%) | 28 (45.2%) | 26 (41.3%) |  |
| Race | Non-White | 33 (26.4%) | 16 (25.8%) | 17 (27.0%) | 1 |
|  | White | 92 (73.6%) | 46 (74.2%) | 46 (73.0%) |  |
| Age | <=5 | 79 (63.2%) | 39 (62.9%) | 40 (63.5%) | 1 |
|  | > 5 | 46 (36.8%) | 23 (37.1%) | 23 (36.5%) |  |
| Vital Status | Alive | 75 (60.0%) | 39 (62.9%) | 36 (57.1%) | 0.635 |
|  | Dead | 50 (40.0%) | 23 (37.1%) | 27 (42.9%) |  |
| Stage | I | 16 (12.8%) | 10 (16.1%) | 6 (9.52%) | 0.738 |
|  | II | 49 (39.2%) | 23 (37.1%) | 26 (41.3%) |  |
|  | III | 46 (36.8%) | 22 (35.5%) | 24 (38.1%) |  |
|  | IV | 14 (11.2%) | 7 (11.3%) | 7 (11.1%) |  |
| Histologic  Classification of Primary Tumor | DAWT | 42(33.6%) | 18(29.03%) | 24(38.1%) | 0.3771 |
|  | FHWT | 83(66.4%) | 44(70.97%) | 39(61.9%) |  |

Notes: FHWT, Favorable Histology Wilms Tumor. DAWT, Diffuse Anaplastic Wilms Tumor. A *p*-value less than 0.05 was considered statistically significant.

**Supplementary Figure 1
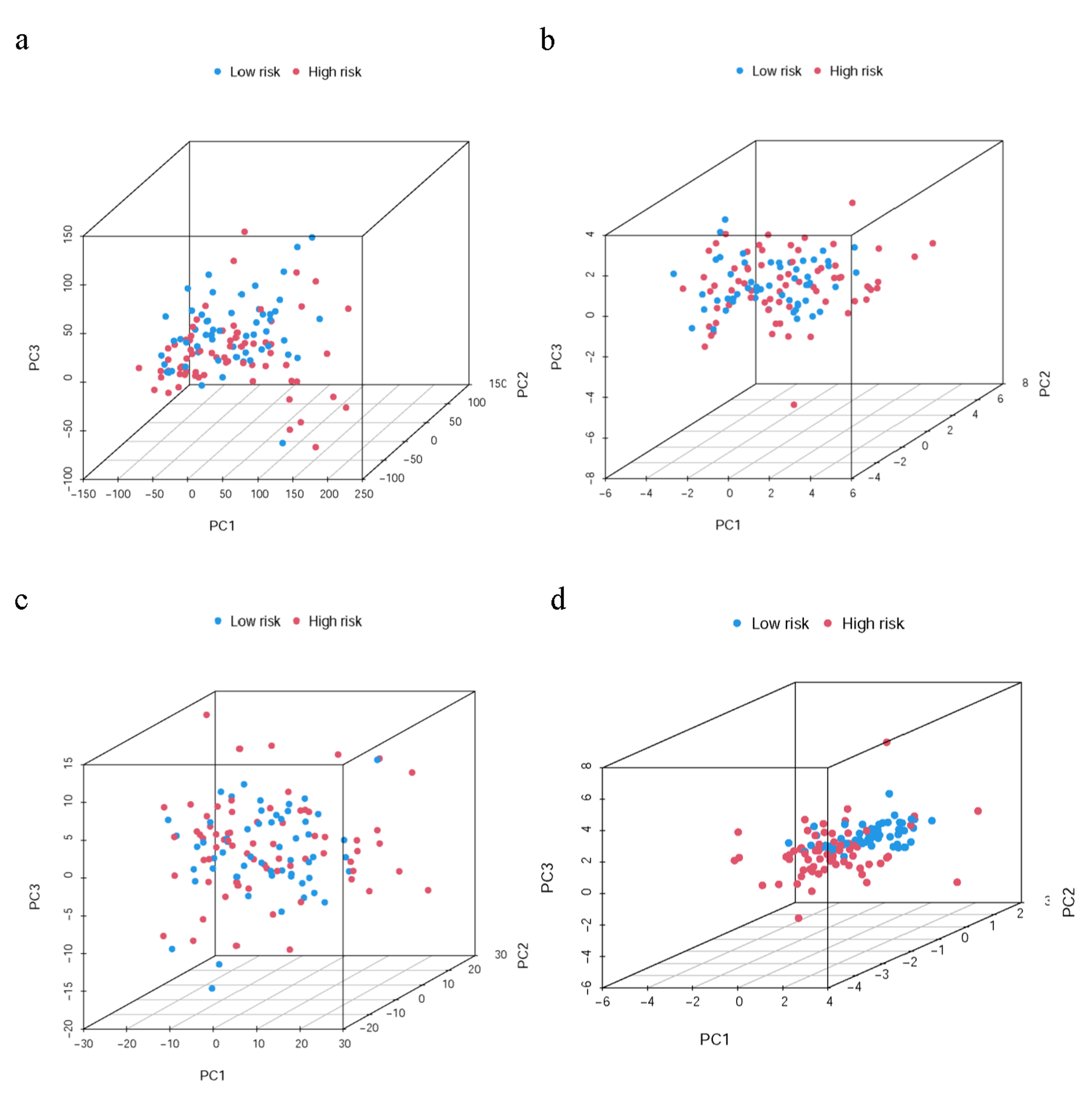
**

**Supplementary Figure 2
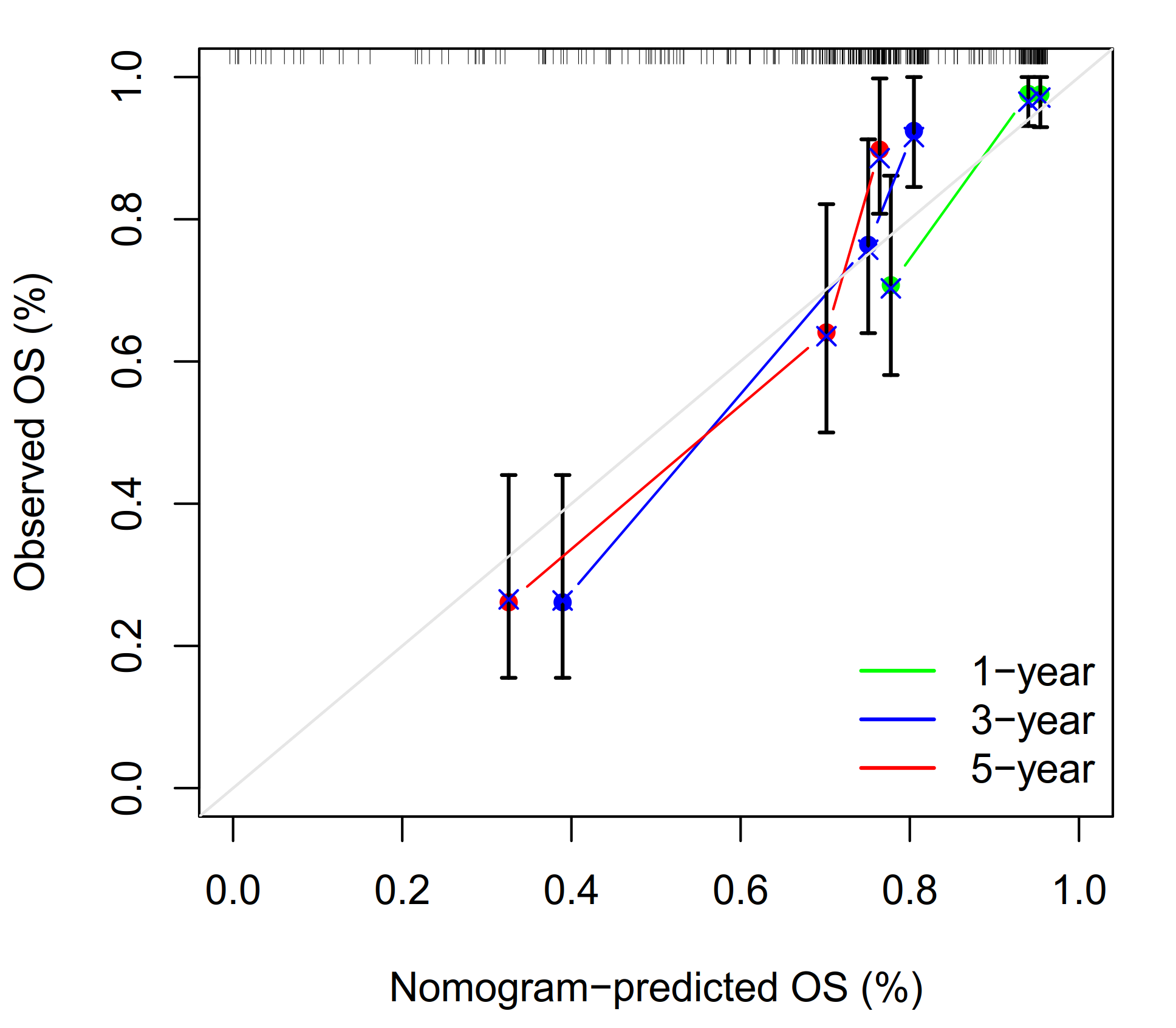
**

**Supplementary Figure 3**

**
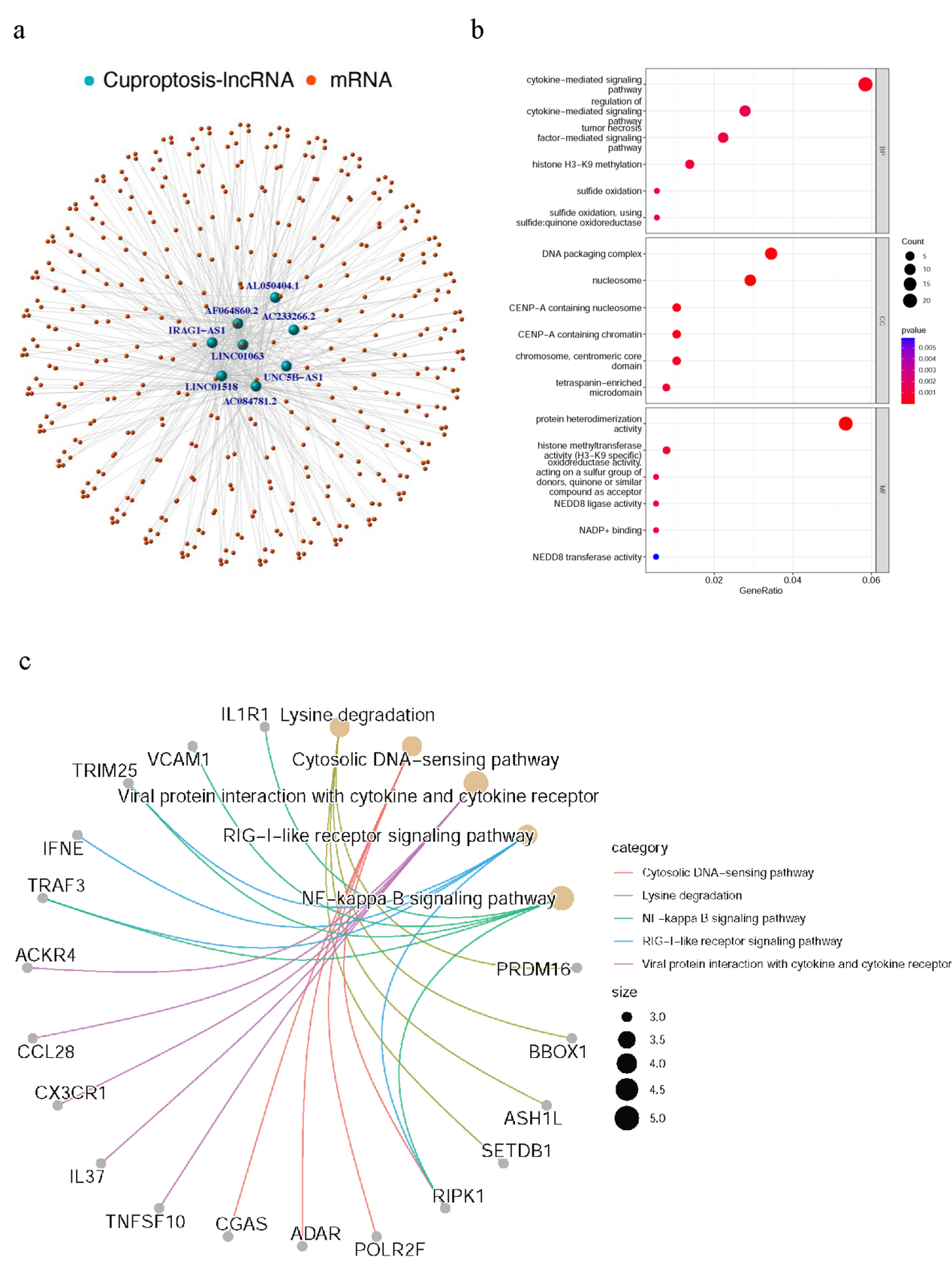
**
